# Abortion, Treatment of

**Published:** 1875-02

**Authors:** Paul F. Munde


Treatment of Abortion.—* * * Hoening recommends to express the ovum, either entire or in part, if the fœtus be already removed, by means of bi-manual compression, two fingers of one hand being introduced into the vagina and passed as far up as possible into the fornix vaginæ, and the other grasping the uterus through the abdominal parietes, thus firmly compressing the organ between the fingers of both hands and slowly and surely expelling its contents. If the uterus is anteverted or anteflexed, as is usual during the earlier months of pregnancy, the two fingers should be passed into the anterior cul-de-sac, or the corpus uteri may be firmly pressed against the symphysis pubis by the external hand alone (the bladder having been emptied;) if the uterus is retroflexed, the two internal fingers go behind the cervix. The relaxation of the abdominal parietes in multiparæ (in whom, as has been statistically shown, most abortions occur,) usually renders the seizure of the uterus by the external hand an easy matter. The facility and rapidity with which the expression of the ovum is accomplished is, according to Hoening, surprising and gratifying, as also the absence of subsequent hemorrhage or puerperal trouble. The pressure on the uterus need not and never should be sufficient to do harm.

Before reading this paper, I happened to meet with a case of miscarriage, in which I succeeded in expressing the retained placenta by bi-manual compression. Since then, during the past summer, I employed the manipulation in two cases, both abortions in the fourth month, in consequence of retroversion, with the most complete success. Passing two fingers into the posterior cul-de-sac, and grasping the uterus firmly with the other hand from without, I immediately felt the uterine contents slip out towards the cervix, and pressed them entirely into the vagina by passing the two fingers behind the cervix forwards, towards the external os. Both ova were expelled into the vagina entire, the membranes of one of them rupturing as it left the os. Both women made easy and rapid recoveries.

Of course it is essential that the cervix be sufficiently dilated before the measure is attempted. In my two last cases I accomplished the dilatation and at the same time controlled the hemorrhage by the introduction of the colpeurynter for several hours. The uterine contractions attending the dilatation of the os will generally loosen the adhesions of the ovum to the uterus sufficiently to allow of its easy expulsion.

The advantage of this *vis a tergo* over the introduction of the speculum, the forceps and the curette, to say nothing of the old method of passing the finger into the uterus, is obvious. I trust this communication may contribute to the general adoption of this most excellent practice. PAUL F. MUNDE, M.D.

